# Subtyping patients with heroin addiction at treatment entry: factor derived from the Self-Report Symptom Inventory (SCL-90)

**DOI:** 10.1186/1744-859X-9-15

**Published:** 2010-04-13

**Authors:** Icro Maremmani, Pier Paolo Pani, Matteo Pacini, Jacopo V Bizzarri, Emanuela Trogu, Angelo GI Maremmani, Gilberto Gerra, Giulio Perugi, Liliana Dell'Osso

**Affiliations:** 1 'Vincent P Dole' Dual Diagnosis Unit, 'Santa Chiara' University Hospital, Department of Psychiatry, NPB, University of Pisa, Pisa, Italy; 2 AU-CNS, 'From Science to Public Policy' Association, Pietrasanta, Lucca, Italy; 3 'G De Lisio', Institute of Behavioural Sciences, Pisa, Italy; 4 Social-Health Direction, Health District 8 (ASL 8), Cagliari, Italy; 5 Drug Addiction Unit, Bolzano, Italy; 6 Global Challenges Section, Human Security Branch, Division for Operations, United Nations Office on Drugs and Crime, Vienna

## Abstract

**Background:**

Addiction is a relapsing chronic condition in which psychiatric phenomena play a crucial role. Psychopathological symptoms in patients with heroin addiction are generally considered to be part of the drug addict's personality, or else to be related to the presence of psychiatric comorbidity, raising doubts about whether patients with long-term abuse of opioids actually possess specific psychopathological dimensions.

**Methods:**

Using the Self-Report Symptom Inventory (SCL-90), we studied the psychopathological dimensions of 1,055 patients with heroin addiction (884 males and 171 females) aged between 16 and 59 years at the beginning of treatment, and their relationship to age, sex and duration of dependence.

**Results:**

A total of 150 (14.2%) patients with heroin addiction showed depressive symptomatology characterised by feelings of worthlessness and being trapped or caught; 257 (24.4%) had somatisation symptoms, 205 (19.4%) interpersonal sensitivity and psychotic symptoms, 235 (22.3%) panic symptomatology, 208 (19.7%) violence and self-aggression. These dimensions were not correlated with sex or duration of dependence. Younger patients with heroin addiction were characterised by higher scores for violence-suicide, sensitivity and panic anxiety symptomatology. Older patients with heroin addiction showed higher scores for somatisation and worthlessness-being trapped symptomatology.

**Conclusions:**

This study supports the hypothesis that mood, anxiety and impulse-control dysregulation are the core of the clinical phenomenology of addiction and should be incorporated into its nosology.

## Background

Patients with substance use disorder (SUD) have increased levels of comorbidity with psychiatric disorders, specifically with mood, anxiety and other impulse-control, imbalance-related disorders [1,2]. Moreover, a higher frequency than that expected on the basis of chance in the association with psychotic disorders has been ascertained [3-10].

The relationship between substance abuse/use/dependence and other psychiatric disorders is a complex one. Theoretically, four explanations are available: the first that the presence of a mental disorder causes or facilitates the manifestation of addiction; the second that substance use disorders elicit the onset of other mental disorders; the third that the underlying causes of substance use and of other psychiatric disorders may be the same, and the fourth that factors linked to sampling, selection of instruments for diagnosis, investigation, and analysis could have led to an incorrect estimation of comorbidity [11].

The current evidence supports each of these possibilities as contributing, to differing degrees, in determining the clinical presentations of comorbidity in addicted individuals. However, even if the existing literature has explored the correlations between substance use and different areas of psychopathology, and put forward hypotheses about the mechanisms that trigger substance use and/or psychopathology, it has left unexplored an extensive grey area pertinent to the question of whether some of the symptoms usually exhibited by addicted people, especially in the domains of mood, anxiety and impulse control, actually belong to addiction or to comorbid psychiatric disorders [11]. This is a central question since, before asking what comes first (addiction or another psychiatric condition), the problem of the real independence of symptoms, or of close linkage between the psychiatric symptoms and the central symptoms of addiction that appear together with it needs to be solved.

In fact, the application of the classic model of psychiatric comorbidity in the field of addiction has been the target of criticisms focused on issues such as the high frequency of this association, which raises the question of the independence of the two conditions, the difficulty of disentangling supposedly independent psychiatric symptoms and syndromes from the core psychopathology of addiction, and the overlap between the biological substrates and the neurophysiology of addictive processes and psychiatric symptoms associated with addiction [11,12]. On these bases a unified perspective has been proposed, foreseeing the inclusion of symptoms of the anxiety, mood and impulse-control domains in the psychopathology of addiction, and considering too those symptoms and syndromes that are below the threshold set for a defined additional mental disorder, even if they heavily condition the everyday life of patients and often require interventions [11,12].

Given this background, and the consequent uncertainty in the correct classification of symptomatology as being intrinsic to the addictive disorder or as due to comorbidity, it seems best to try to approach the psychopathology of addicts by starting from a low inference level rooted in the symptoms expressed by patients rather than starting from a pre-established syndromic level such as that of the Diagnostic and Statistical Manual of Mental Disorders (DSM) nosography. From this foundation, the identification, in addicts, of what are probably composite psychological/psychiatric dimensions resulting from the spontaneous association between symptoms should be considered a priority.

In this article we have tried to subtype patients with heroin dependence on the basis of their answers to the Self-Report Symptom Inventory (SCL-90) questionnaire.

## Methods

### Sample

Inclusion criteria comprised a diagnosis of heroin addiction according to DSM-IV criteria and duration of illness of at least 1 year.

The sample consisted of 1,055 subjects, evaluated at their treatment entry. Data came from the Pisa addiction dataset: a database including anonymous individual information originally collected for clinical or other research purposes. Mean age was 30 ± 7 years (range 16 to 59), 884 (83.8%) were male, 133 (12.6%) had a low educational level (less than 8 years), 691 (65.5%) had never been married, 483 (45.8%) were unemployed and 25 (2.4%) were unable to work due to health impairment. Among those employed, 295 (28.0%) had a 'white collar' job and 276 (26.2%) a 'blue collar' job. Mean duration of addiction was 7.20 ± 6.0 years. A total of 502 (47.6%) had been addicted for less than 5 years, 272 (25.8%) between 5 and 10 years, 152 (14.4%) between 10 and 15 years, 100 (9.5%) between 15 and 20 years, 29 (2.7%) between 20 and 28 years. All these patients were Italians, and were only included once in the sample. In all, 170 (16.1%) began treatment for the first time.

### Instruments

Developed by Derogatis and colleagues [13], the SCL-90 is made up of 90 items, each rated on a 5-point scale of distress. These items are clustered in nine dimensions. 'Somatisation' reflects distress arising from perceptions of bodily dysfunction. Complaints focused on cardiovascular, gastrointestinal, respiratory and other systems with strong autonomic mediation have been included. Headaches, backaches, and pain and discomfort localised in the gross musculature are additional components, as are other somatic equivalents of anxiety. 'Obsessive-Compulsive' reflects behaviours that are closely identified with the clinical syndrome of the same name. The focus of this criterion is on thoughts, impulses and actions that are experienced as unremitting and irresistible by the individual but are of an ego-alien or unwanted nature. Behaviours indicative of a more general cognitive difficulty (for example, 'mind going blank', 'trouble remembering') also load on this dimension. 'Interpersonal Sensitivity' focuses on feelings of personal inadequacy and inferiority, particularly by comparison with other individuals. Self-deprecation, feelings of uneasiness, and marked discomfort during interpersonal interactions are characteristics of people showing high levels for this dimension. Feelings of self-consciousness and negative expectations regarding interpersonal communications are further typical sources of distress. 'Depression' reflects a broad range of the concomitants of the clinical depressive syndrome. Symptoms of dysphoric affect and mood are represented, as are signs of withdrawal of interest in life events, lack of motivation, and loss of vital energy. This dimension mirrors feelings of hopelessness and futility, as well as other cognitive and somatic correlates of depression. Several of the items included have to do with thoughts of death and suicidal ideation. 'Anxiety' subsumes a set of symptoms and experiences usually associated clinically with a high degree of manifest anxiety. General indicators such as restlessness, nervousness, and tension are included here, as are additional somatic c signs (for example, 'trembling'). Scales measuring free-floating anxiety and panic attacks are an integral aspect of this dimension, and an item on feelings of dissociation is included. 'Hostility' is organised around three categories of hostile behaviour: thoughts, feelings, and actions. Items range from feelings of annoyance and urges to break things, to arguments and uncontrollable temper outbursts. 'Phobic Anxiety' reflects symptoms that have been observed with a high incidence in conditions termed phobic anxiety state or agoraphobia. Fears of a phobic nature oriented towards travel away from home, open spaces, crowds, or public places and means of transport are represented by this parameter. In addition, several scales representing social phobic behaviour have been included. 'Paranoid Ideation' derives from the notion that paranoid behaviour is best considered from a syndromal point of view. Projective ideation of hostility, suspiciousness, centrality, delusions, loss of autonomy, and grandiosity as cardinal paranoid characteristics are assessed within the limitations imposed by a self-report format. 'Psychoticism' represents florid, acute symptomatology, as well as behaviours typically viewed as more oblique, less definitive, indicators of psychotic processes. Four items reflect Schneiderian first rank symptoms of schizophrenia: auditory hallucinations, thought broadcasting, external thought control, and external thought insertion. In addition, secondary signs of psychotic behaviour, as well as indications of a schizoid life style, are represented too. Global scores for SCL-90 items are Total SCL-90 score (sum of all items), the number of items rated positively (PST), and the positive symptom distress index (PSDI), which is calculated by dividing the sum of all items by the score for PST.

### Data analysis

An exploratory factor analysis was performed on the 90 SCL items. The ratio of patients/items (11:1) is high enough to authorise this analysis because it is higher than the recommended 10:1 ratio. Factors were extracted by using a principal component analysis (PCA; type 2) and then rotating this orthogonally to achieve a simple structure.

This simplification is equivalent to maximising the variance of the squared loading in each column. To limit the factor number, the criterion used was an eigenvalue >1.5. Items loading with absolute values >0.40 were used to describe the factors. This procedure makes it possible to minimise the crossloadings of items on factors. In order to make factor scores comparable, they were standardised into z scores. All the subjects were assigned to one of five different subtypes on the basis of the highest factor score achieved (dominant SCL-90 factor). This procedure gives the opportunity to classify subjects on the basis of the highest symptomatological cluster. In this way it is possible to solve the problem of identifying a cut-off point for the inclusion of patients in the different clusters identified.

In order to verify how distinct the subtypes are, we analysed the mean z scores and 95% CI across the factors for each dominant group. We also performed a discriminant analysis by utilising the scores of the five factors to predict membership in each dominant group. Lastly, we compared age, sex and duration of dependence between the various dominant SCL-90 factor groups. Continuous variables were compared between groups by means of one-way ANOVA followed by *post hoc *Student-Newman-Keuls F test or by Kruskal-Wallis test when appropriate, and categorical ones by means of χ^2 ^analysis. All statistical analyses were carried out using SPSS v. 4.0 (SPSS, Chicago, IL, USA).

We did not analyse age and gender correlations with SCL-90 before the factor analysis because SCL-90 is a symptom scale and not a psychological test. As a result, the scale response is not affected by age and gender but by the level of severity of psychiatric disorders. Factor analysis is used to summarise the empirical correlations of SCL-90 items into psychopathological dimensions. Therefore, age and gender do not enter into factor analysis. However, exploring the relationship between the psychopathological dimensions derived from factor analysis and age and gender is of clinical interest, because it may provide useful hints about gender-specific and age-specific psychopathological profiles in patients with addiction.

Additionally, SCL-90 global scores were left unanalysed. Our choice was to focus on differential psychopathological profiles, and these tend to be obscured when global scores are used.

## Results

### Factor analysis

Using an exploratory PCA of the 90 items of the SCL-90, a 5-factor solution was identified (Table 1). A total of 77 items with a loading >0.40 were retained. We named factors on the basis of items with the highest loadings. The first factor reflected a depressive 'worthlessness and being trapped' dimension; this accounted for 29.9% of the variance. The second factor, accounting for 4.2% of the variance, picked out a 'somatisation' dimension. The third factor identified a 'sensitivity-psychoticism' dimension; this accounted for 3.0% of the total variance. Panic symptoms loaded on the fourth factor, 'panic anxiety', accounted for 2.15% of the total variance. The last, fifth factor singled out a 'violence-suicide' dimension, which accounted for 2.0% of the total variance. Overall, the five factors accounted for 37.8% of the variance of the items.

**Table 1:** Factor loading of the 77 Self-Report Symptom Inventory (SCL-90) items retained in the principal component analysis (PCA)

SCL-90 item and no.	Worthlessness-being trapped	Somatic symptoms	Sensitivity-psychoticism	Panic-anxiety	Violence-suicide
02. Nervousness or shakiness inside					0.42

03. Unwanted thoughts, words, or ideas that won't leave your mind	0.41				

04. Faintness or dizziness				0.48	

05. Loss of sexual interest or pleasure	0.44				

07. The idea that someone else can control your thoughts			0.51		

10. Worried about sloppiness or carelessness	0.48				

11. Feeling easily annoyed and irritated		0.40			

12. Pains in heart or chest		0.43			

13. Feeling afraid in open spaces or on the streets				0.60	

14. Feeling low in energy or slowed down		0.59			

15. Thoughts of ending your life					0.48

17. Trembling		0.46			

19. Poor appetite		0.44			

22. Feeling of being trapped or caught	0.68				

23. Suddenly scared for no reason				0.41	

24. Temper outbursts that you could not control					0.60

25. Feeling afraid to go out of your house alone				0.56	

26. Blaming yourself for things	0.43				

27. Pains in lower back		0.61			

28. Feeling blocked in getting things done	0.53				

29. Feeling lonely	0.66				

30. Feeling blue	0.66				

31. Worrying too much about things	0.47				

32. Feeling no interest in things	0.63				

33. Feeling fearful				0.44	

34. Your feelings being easily hurt	0.45				

35. Other people being aware of your private thoughts			0.58		

36. Feeling others do not understand you or are unsympathetic			0.54		

37. Feeling that people are unfriendly or dislike you			0.56		

38. Having to do things very slowly to ensure correctness			0.46		

39. Heart pounding or racing		0.46			

40. Nausea or upset stomach		0.62			

41. Feeling inferior to others	0.57				

42. Soreness of your muscles		0.73			

43. Feeling that you are watched or talked about by others			0.59		

44. Trouble falling sleep		0.62			

45. Having to check and double check what you do			0.47		

46. Difficulty making decisions	0.54				

47. Feeling afraid to travel on buses, subways, or on trains				0.53	

48. Trouble getting your breath		0.46			

49. Hot or cold spells		0.69			

50. Having to avoid certain things, places, or activities because they frighten you				0.42	

51. Your mind going blank	0.44				

52. Numbness or tingling in parts of your body		0.50			

53. A lump in your throat		0.48			

54. Feeling hopeless about the future	0.64				

55. Trouble concentrating	0.52				

56. Feeling weak in parts of your body		0.62			

57. Feeling tense or keyed up	0.43				

58. Heavy feelings in your arms or legs		0.70			

59. Thoughts of death or dying					0.47

61. Feeling uneasy when people are watching or talking about you			0.50		

62. Having thoughts that are not your own			0.51		

63. Having urges to beat, injure, or harm someone					0.54

64. Waking up early in the morning		0.52			

66. Sleep that is restless or disturbed		0.57			

67. Having urges to break or smash things up					0.67

68. Having ideas or beliefs that others do not share			0.41		

69. Feeling very self-conscious with others			0.48		

70. Feeling uneasy in crowds, such as shopping or at a movie			0.42		

71. Feeling everything is an effort	0.52				

72. Spells of terror or panic				0.54	

73. Feeling uncomfortable about eating or drinking in public			0.41		

74. Getting into frequent arguments					0.51

75. Feeling nervous when you are left alone	0.40				

76. Others not giving you proper credit for your achievement			0.45		

77. Feeling lonely even when you are with people	0.60				

78. Feeling so restless you couldn't sit still					0.40

79. Feelings of worthlessness	0.69				

80. Feeling that familiar things are strange or unreal			0.40		

81. Shouting or throwing things					0.70

82. Feeling afraid you will faint in public				0.47	

83. Feeling that people will take advantage of you if you let them			0.46		

86. Feeling pushed to get things done			0.41		

88. Never feeling close to another person	0.50				

89. Feelings of guilt	0.53				

90. The idea that something is wrong with your mind	0.52				

Eingenvalue	26.8	3.78	2.70	2.15	1.85

Variance	29.9	4.2	3.0	2.4	2.0

On the basis of the highest z scores obtained on the five SCL-90 factors (dominant SCL-90 factor) subjects were assigned to five mutually exclusive groups. The group whose dominant was 'worthlessness and being trapped' comprised 150 subjects (14.2%), the group with 'somatisation' as its dominant gathered 257 subjects (24.4%), the group showing 'sensitivity-psychoticism' as its dominant included 205 subjects (19.4%), the group identified by 'panic anxiety' as its dominant numbered 235 subjects (22.3%), and the group whose dominant was 'violence-suicide' group profiled a cluster of 208 subjects (19.7%). These five groups were sufficiently distinct, and failed to reveal any significant overlap. All these patients showed positive scores in their dominant factors only, alongside negative scores in all the others, the only exception being a small number of patients whose dominant was 'worthlessness and being trapped', who recorded a positive score for the 'sensitivity psychoticism' factor (mean = 0.07; 95% CI = -0.06 to 0.19) This finding was confirmed by the discriminant analysis, which indicated a percentage of correctly classified 'grouped' cases as high as 95.26%.

The main factor (worthlessness, feeling trapped) brings together depressive, obsessive-compulsive and psychotic symptoms. Treatment-seeking addicts who display depressed mood usually report feelings of uselessness and the feeling of being trapped in a corner. These patients feel abandoned, sad, with no goal or interest; they are excessively preoccupied with difficulties, and report feelings of guilt, while experiencing a low or zero sexual drive, too. Obsessive-compulsive symptoms include difficulties in making decisions, completing a task and concentrating, along with worries about one's ineptitude, an 'empty mind' sensation and an incapacity to dominate one's thoughts. Other symptoms, such as the need to check out actions several times or act slowly so as to avoid making mistakes, are not featured. Compulsions and memory impairment do not appear in any factor. Thought disorders consist of feeling alone even when with other people, the thought that one's mind is not working properly, while never feeling really close to others. Lastly, these subjects report a feeling of inferiority, are easily hurt (interpersonal sensitivity), do not like being alone (phobic anxiety) and often feel nervous and upset ('free' anxiety). On the whole, this factor is essentially made up of depressive, obsessive and psychotic features, dominated by feelings of uselessness and of being trapped in a corner.

The second factor (somatisation) is distinguished by a number of somatic and anxious elements, which are usually a feature of opiate withdrawal. The patient complains of muscle aches, back pain, heavy legs and arms, weakness and tiredness, loss of sensitivity and paraesthesia somewhere in the body. Hot flushes and cold shivers are possible too, as well as nausea and stomach ache. Sleep is disturbed and broken up, while getting to sleep is difficult. Patients wake up early at dawn and cannot get back to sleep. They report a sensation of choking, or of being breathless; they may tremble, are aware of their heart beating, or even of chest pain. Appetite is low. Interpersonal sensitivity is heightened, so that they are easily annoyed and irritated.

The third factor features sensitivity and psychoticism. Patients have the impression that others stare at them and speak about them, may do something against them or exploit them with unpredictable consequences. They think they are not respected by their workmates or are disapproved of because of their own views. They get the impression that others do not sympathise with them or approve of their behaviour, or even show explicit hostility towards them. They feel uneasy when they find other people staring at them or simply in speaking with acquaintances, or may even feel threatened when others are there in the same room. They feel uncomfortable in open or crowded spaces, or when doing things in a group (for example, eating). These behaviours may be defined as psychotic as long as the patient is convinced that others control or influence their thoughts, in some cases actually being identified as imposed from outside that individual's mind. Obsessive-compulsive features of a checking type, or taking a lot of time in doing things out of a fear of making mistakes, may also be part of the picture. Lastly, there may be feelings of estrangement and detachment from reality, with the impression that common and familiar things no longer belong to them.

The fourth factor (panic anxiety) can be summed up as agoraphobia, a fear of going around alone, episodes of critical anxiety, fear of travelling by bus, train or subway, sensations of fainting, dizziness or fear of feeling sick or upset in front of other people. Generalised fear is a feature, with the need to avoid certain things, places or activities in order to prevent panicking.

The fifth factor (violence-suicide) includes violent acting outs and features of self-directed aggressiveness. Patients have moments when they cry or throw objects with the aim of breaking them or smashing them into pieces, and suffer from outbursts of rage. They often get into arguments and feel the urge to push, hurt or beat up others. Side by side with all this, they have suicidal thoughts, or longings for death, are upset, excited or restless, and find it hard to stay seated or lie down for any length of time.

### Characteristics of patients with heroin addiction in the five groups

The female/male ratio was 1:4.5 for patients in the 'worthlessness and being trapped' group (group 1), 1:6.4 for 'somatisation' (group 2), 1:7.1 for 'sensitivity-psychoticism' ones (group 3), 1:5 for 'panic anxiety' (group 4) and 1:3.7 for 'violence-suicide' (group 5). These differences were not statistically significant (χ^2 ^= 6.83 *P *= not significant).

Length of dependence (years) was 8 ± 6 years for group 1, 8 ± 6 for group 2, 7 ± 6 for group 3, 7 ± 6 for group 4 and 7 ± 6 for group 5 patients. No significant differences were observed (Kruskal-Wallis test = 5.69 *P *= not significant).

Group 1 patients were 31 ± 7 years old; group 2 patients were 31 ± 7 years old; group 3 patients were 29 ± 7 years old; group 4 patients were 30 ± 7 years old and group 5 patients were 29 ± 6 years old. Patients belonging to group 2 did not differ from those belonging to group 1 or to group 4 patients, but, with statistical significance, were older than patients belonging to group 3 and group 5 (F = 4.79 *P *< 0.01). Younger heroin addicts displayed higher scores for violence-suicide, sensitivity and panic anxiety symptomatology. Older heroin addicts were distinguished by higher scores for somatisation and worthlessness-being trapped symptomatology.

## Discussion

### Factor analysis

The presence of a depressive dimension factor in opioid addicts at treatment entry is not surprising. It can be justified by psychological/psychiatric conditions preceding or following substance abuse and dependence. Precursors such as sensation-seeking, impulsiveness, behavioural disinhibition, hyperthymic and cyclothymic temperaments, typically framed in the bipolar mood spectrum, have all been considered predictive of subsequent addictive behaviour [14-21]: they are all candidates ranking as possible facilitators of substance encounter and escalation to addiction [12,17,22,23]. Moreover, mood alterations can follow substance abuse and dependence. Besides depression, anxiety, and dysphoric mood accompanying opioid, stimulant, and alcohol or sedative withdrawal, the persistence of a depressive state related to repeated substance use has been observed with alcohol and other substances [24-28] and one hypothesis put forward is that of a reward deficiency syndrome, with anhedonia and a difficulty in deriving pleasure from non-drug related stimuli both prominent [29].

As stated in the Introduction, the association between mood disorders and addiction may involve such a close interaction at neurobiological levels between predisposing factors, addictive processes and addictive consequences, that the attempt to clinically distinguish between addictive-related or independent depression may turn out to be little more than an inconclusive theoretical exercise [11]. The depressive condition experienced by opioid addicts when asking for treatment may originate in a multifactorial interaction which gives rise to the particularities of clinical presentation, marked out by several depressive features, the most prominent of which are feelings of uselessness and of being trapped in a corner.

The second psychological/psychiatric dimension, shown by opioid addicts on entering treatment can be recognised from somatic symptoms. These are consistent with those that are observed within the opiate withdrawal syndrome and are associated with anxiety. Anxiety is again a major feature in the fourth dimension resulting from factorial analysis, in the form of panic anxiety-related symptoms. Anxiety and panic anxiety may be linked with the withdrawal syndrome. The pathophysiology of withdrawal actually overlaps with that of panic disorder, as noradrenergic circuitry around the locus coeruleus is involved in both cases: the cognitive aspect (substance deficiency vs. fear of dying or losing control) usually makes the difference, but most addicts often mistake panic symptoms for withdrawal, however unlikely this may be in given circumstances, or develop the conviction that substance use during withdrawal will prevent them from undergoing potentially dangerous arousal, in the context of a panic-related cognitive conditioning.

However, anxiety is not peculiar to opiate withdrawal, and other determinants of anxiety disorders in addicts cannot be skipped. In fact, a substance-associated nature has been indicated in 20% of panic cases, 25% of social phobias, 40% of obsessive-compulsive disorders, and 50% of agoraphobia [24,30].

The third psychological/psychiatric dimension shown by opioid addicts on entering treatment is characterised by sensitivity and psychoticism. The relationship between psychotic symptoms and substance use has been widely investigated. The most typical case of psychosis in addicted persons is the appearance of a schizophrenia-like syndrome in chronic stimulant abusers. Psychotic symptoms are reported in 40% of stimulant abusers [31,32], and in half of the more chronic cocaine users [33,34]. Such symptoms have been observed even in the absence of psychiatric substance-related vulnerability [35] and can, in fact, be induced experimentally [36-41]. Psychotic symptoms may also be associated with cannabinoid use, where the symptoms usually produced by cannabis, such as anxiety, depersonalisation, and derealisation [42], increase and are found associated with hallucinations and delusions [43-46]. Alcohol too has been associated with the appearance of psychotic symptoms both during intoxication and withdrawal [47,48]. However, the prevalence of psychotic disorders in opioid addicts is low. This may depend on a variety of factors, such as the difficulty chronic psychotics have in going through the regular environmental interactions that are made unavoidable by their need for drug supplies, or on the glossing over that derives from the general antidysphoric effects of opioids and long-acting opioids such as methadone in masking the proneness to psychosis of some patients. This last explanation is consistent with the existence of an opiate withdrawal syndrome [49]. In fact, the majority of psychotic subjects who develop opioid addiction are more likely to be diagnosed as suffering from borderline pictures, intermittent psychotic disorders such as bipolar I, or atypical pictures including substance-induced psychosis. Also, given the high rate of current polyabuse of psychotomimetic drugs, such as cannabis, mild psychotic syndromes may be frequent on psychometric grounds, even when underrated on clinical grounds [42-46].

Lastly, the fifth psychological/psychiatric dimension shown by opioid addicts on treatment entry is most easily identified through by violent acting outs and features of self-directed aggressiveness. Aggressiveness and self-injurious behaviour are far from being incompatible, and usually run parallel, as both are supported by impulsiveness, often reflecting the severity of opiate intoxication [50]. The form usually taken by impulsiveness in addicts is connected with their extreme proneness to drug-related stimuli [51-57], but a more general reduction of inhibitory control over impulsiveness in areas of behaviour not directly linked with drug use can be observed. The performance of smokers, alcoholics, cocaine users, and opiate addicts in carrying out behavioural tasks designed to measure impulsiveness, such as the Iowa Gambling Task, Stroop test, and other behavioural inhibition tasks, indicates an increase in the level of impulsiveness [58-65]. The altered response to these tests may easily depend on an underlying, previously active mental disorder or earlier psychic conditions [66-72]. Moreover, data consistent with the direct action of drugs in inducing impulsiveness have been reported for cases of nicotine, alcohol, heroin and cocaine use [58,73-77].

Subjects with impulsive personality structures and earlier involvement in drug use are those who seem to develop the most severe withdrawal syndromes, suggesting that opiate balance and control over aggressiveness share the same roots. Before the onset of addiction, impulsive subjects display proneness to aggression, but also a disposition towards risk taking, drug use included. In the context of drug use, these subjects show a tendency to move more quickly towards quicker transition to tolerance and regular drug use. Once addiction has developed, the two kinds of damage run parallel and mirror the severity of addiction itself, together with the disruptive behaviour associated with drug seeking. Even in the case of impulsiveness, rage and violence, it is often impossible to disentangle earlier psychological/psychiatric conditions from those that follow the effects of substances, addictive processes, psychiatric consequences and their interactions

### Characteristics of patients with heroin addiction in the five groups

Among the sociodemographic variables investigated, the only one that significantly differentiated the groups of patients identified by factor analysis was age: sensitive-psychotic, violent/suicidal and panic addicts proved to be younger. Psychopathological dimensions seem to be unrelated to gender, since the sex ratio does not vary to a significant degree across dimensions. Even the duration of dependence did not differ between dimensions, so that the contribution to the quality of symptoms can be considered similar, and subtypes stand as distinct psychopathological profiles.

## Limitations

Urinalyses were not available for all subjects beyond the knowledge of their actual heroin use status. As a result, interpretation of psychopathology through a polyabuse profile was not possible. However, no current intoxication or withdrawal syndrome was ongoing at the time of questionnaire administration, so that possible positive non-opiate substance use status was subclinical, and, in any case, unknown.

The profiles of all these subjects were based on self-evaluation, but this method of evaluation leaves open possible discordance between self-evaluated psychopathology and observer-related 'objective' evaluation for some SCL90 items. Given that the theoretical option of having 1,055 subjects evaluated in an objective manner by the same interviewer was not feasible, our preference went to patient-related self-evaluation, rather than non-uniform interviewer-related objective rating.

A second limitation is that results can only be considered representative of heroin addicts who apply for treatment, and at time of treatment request. Some symptoms may vary at different stages of the disease, whereas some may be crucial in favouring or impeding treatment requests, so that they may be under/overfeatured in our sample.

No relationship with psychiatric diagnosis was possible on methodological grounds, since data were collected in a cross-sectional way, and diagnostic homogeneity was presumably low, given the heterogeneous sources of data. In Italy, patients may, in fact, resort to psychiatric facilities or local addiction treatment units, but psychiatric diagnosis is not always formulated early in the course of treatment, let alone at treatment entrance. Moreover, the absence of complete urinalyses hampered diagnostic reliability for some of the subjects.

## Concluding remarks

All in all, no unique interpretation can be relied upon unreservedly. On one hand, some symptoms may mirror the severity of chronic heroin intoxication, including pictures of polyabuse of synergic drugs such as depressants or alcohol. On the other hand, some may be no more than a sign of concurrent psychiatric disorders; others may be a consequence of polyabuse patterns, with special reference to mixed polyabuse. From a different viewpoint, severity may, with the same factors, distinguish between intoxication-related pictures and dual diagnosis, so that patterns of severe behavioural disorder probably point to dual diagnosis, whereas milder abnormalities may be predictable as the outcome of continued drug abuse. For mentally ill patients, or patients with a subclinical disposition to mental disorders, drug abuse may play an amplifying role, leading to full blown or more severe clinical pictures. The combination between subthreshold syndromes and heroin-related amplification may lead to what was originally labelled as an addictive personality, which has mostly been derived from the observation of people who have already undergone chronic exposure to substances and developed addictive diseases. In conclusion, the interaction between the different factors named above should be considered in explaining the presence of psychopathology in opioid addicts who request treatment. Again, the hypothesis that mood, anxiety and impulse-control dysregulation is at the very core both of the origins and the clinical phenomenology of addiction should be considered, as well as the crucial role played by psychiatric manifestations as addiction progresses [11].

More research is needed to confirm our results, to clarify differences between the groups assigned to the five psychopathological dimensions whose profiles have been set out above, and to predict which symptoms will respond to simple anticraving treatment and which need to be targeted separately. In other words, it should be known which heroin addicts stick to the predictable stereotype and which belong to special categories to be handled with specific treatment choices. On grounds of treatment addiction, some symptoms (both for stereotype cases and for special populations) may be predictive of short-term relapse, so that symptomatological screening may provide physicians with a simple instrument for psychopathologically-oriented relapse prevention

## Competing interests

The authors declare that they have no competing interests.

## Authors' contributions

IM, PPP, MP AGIM conceived of the study, and participated in its design and coordination. IM performed the statistical analysis. JVB, ET, GG, GP and LDO revised the first draft and provided suggestions All authors read and approved the final manuscript.

## References

[B1] Regier DA Farmer ME Rae DS Locke BZ Keith SJ Judd LL Goodwin FK Comorbidity of mental disorders with alchool and other drug abuse JAMA 1990 19 2511 2518 10.1001/jama.264.19.2511 2232018

[B2] Kessler RC McGonagle KA Zhao S Nelson CB Hughes M Eshleman S Wittchen HV Kendler HS Lifetime and 12-month prevalence of DSM-III-R psychiatric disorders in the United States: results from the National Comorbidity study Arch Gen Psychiatry 1994 51 8 19 8279933 10.1001/archpsyc.1994.03950010008002

[B3] Clerici M Carta I Cazzullo CL Substance abuse and psychopathology: a diagnostic screening of Italian narcotic addicts Soc Psychiatry Psychiatr Epidemiol 1989 24 219 226 10.1007/BF02351822 2502865

[B4] Pozzi G Bacigalupi M Tempesta E Comorbidity of drug dependence and other mental disorders: a two-phase study of prevalence at outpatient treatment centres in Italy Drug Alcohol Depend 1997 46 69 77 10.1016/S0376-8716(97)00042-2 9246554

[B5] Milby JB Sims MK Khuder S Schumacher JE Huggins N McLellan AT Woody G Haas N Psychiatric comorbidity: prevalence in methadone maintenance treatment Am J Drug Alcohol Abuse 1996 22 95 107 10.3109/00952999609001647 8651147

[B6] Brooner RK King VL Kidorf M Schmidt CW George MD Bigelow GE Among treatment-seeking opioid abusers Arch Gen Psychiatry 1997 54 71 80 9006403 10.1001/archpsyc.1997.01830130077015

[B7] Mason BJ Kocsis JH Melia D Khuri ET Sweeney J Wells A Borg L Millman RB Kreek MJ Psychiatric comorbidity in methadone maintained patients J Addict Dis 1998 17 75 89 10.1300/J069v17n03_07 9789161

[B8] Franken IH Hendriks VM Screening and diagnosis of anxiety and mood disorders in substance abuse patients Am J Addict 2001 10 30 39 10.1080/105504901750160448 11268826

[B9] Ross HE Swinson R Doumani S Larkin EJ Diagnosing comorbidity in substance abusers: a comparison of the test-retest reliability of two interviews Am J Drug Alcohol Abuse 1995 21 167 185 10.3109/00952999509002686 7639204

[B10] Ross J Teesson M Darke S Lynskey M Ali R Ritter A Cooke R The characteristics of heroin users entering treatment: findings from the Australian treatment outcome study (ATOS) Drug Alcohol Rev 2005 24 411 418 10.1080/09595230500286039 16298835

[B11] Pani PP Maremmani I Trogu E Gessa GL Ruiz P Akiskal HS Delineating the psychic structure of substance abuse and addictions: Should it include anxiety, mood and impulse-control dysregulation? J Affect Disord 2010 122 185 197 10.1016/j.jad.2009.06.012 19584019

[B12] Maremmani I Perugi G Pacini M Akiskal H Toward a unitary perspective on the bipolar spectrum and substance abuse: opiate addiction as a paradigm J Affect Disord 2006 93 1 12 10.1016/j.jad.2006.02.022 16675028

[B13] Derogatis LR Lipman RS Rickels K The Hopkins Symptom Checklist (HSCL) - a self report symptom inventory Behav Sci 1974 19 1 16 10.1002/bs.3830190102 4808738

[B14] Akiskal HS Mallya G Criteria for the 'soft' bipolar spectrum: treatment implications Psychopharmacol Bull 1987 23 68 73 3602332

[B15] Placidi GF Signoretta S Liguori A Gervasi R Maremmani I Akiskal HS The semi-structured affective temperament interview (TEMPS-I). Reliability and psychometric properties in 1010 14-26-year-old students J Affect Disord 1998 47 1 10 10.1016/S0165-0327(97)00122-5 9476738

[B16] Sher KJ Bartholow BD Wood MD Personality and substance use disorders: a prospective study J Consult Clin Psychol 2000 68 818 829 10.1037/0022-006X.68.5.818 11068968

[B17] Perugi G Frare F Madaro D Maremmani I Akiskal HS Alcohol abuse in social phobic patients: is there a bipolar connection? J Affect Disord 2002 68 33 39 10.1016/S0165-0327(00)00316-5 11869780

[B18] Maremmani I Pacini M Lubrano S Lovrecic M Perugi G Dual diagnosis heroin addicts. The clinical and therapeutic aspects Heroin Addict Relat Clin Probl 2003 5 7 98

[B19] Akiskal HS Akiskal KK Haykal RF Manning JS Connor PD TEMPS-A: progress towards validation of a self-rated clinical version of the Temperament Evaluation of the Memphis, Pisa, Paris, and San Diego Autoquestionnaire J Affect Disord 2005 85 3 16 10.1016/j.jad.2004.12.001 15780671

[B20] Akiskal HS Mendlowicz MV Jean-Louis G Rapaport MH Kelsoe JR Gillin JC Smith TL TEMPS-A: validation of a short version of a self-rated instrument designed to measure variations in temperament J Affect Disord 2005 85 45 52 10.1016/j.jad.2003.10.012 15780675

[B21] Gerra G Angioni L Zaimovic A Moi G Bussandri M Bertacca S Santoro G Gardini S Caccavari R Nicoli MA Substance use among high-school students: relationships with temperament, personality traits, and parental care perception Subst Use Misuse 2004 39 345 367 10.1081/JA-120028493 15061565

[B22] Akiskal HS Maser JD Zeller PJ Endicott J Coryell W Keller M Warshaw M Clayton P Goodwin F Switching from unipolar to bipolar II. An 11-year prospective study of clinical and temperamental predictors in 559 patients Arch Gen Psychiatry 1995 52 114 123 7848047 10.1001/archpsyc.1995.03950140032004

[B23] Camacho A Akiskal HS Proposal for a bipolar-stimulant spectrum: temperament, diagnostic validation and therapeutic outcomes with mood stabilizers J Affect Disord 2005 85 217 230 10.1016/j.jad.2003.10.014 15780692

[B24] Schuckit MA Tipp JE Bucholz KK Nurnberger JI Hesselbrock Crowe RR Kramer J The life-time rates of three major mood disorders and four major anxiety disorders in alcoholics and controls Addiction 1997 92 1289 1304 10.1111/j.1360-0443.1997.tb02848.x 9489046

[B25] Schuckit MA Tipp JE Bergman M Reich W Hesselbrock VM Smith TL Comparison of induced and independent major depressive disorders in 2,945 alcoholics Am J Psychiatry 1997 154 948 957 9210745 10.1176/ajp.154.7.948

[B26] Gilder DA Wall TL Ehlers CL Comorbidity of select anxiety and affective disorders with alcohol dependence in Southwest California Indians Alcohol Clin Exp Res 2004 28 1805 1813 10.1097/01.ALC.0000148116.27875.B0 15608596

[B27] Conner KR Sörensen S Leonard KE Initial depression and subsequent drinking during alcoholism treatment J Stud Alcohol 2005 66 401 406 16047530 10.15288/jsa.2005.66.401

[B28] Brook DW Brook JS Zhang C Cohen P Whiteman M Drug use and the risk of major depressive disorder, alcohol dependence, and substance use disorders Arch Gen Psychiatry 2002 59 1039 1044 10.1001/archpsyc.59.11.1039 12418937

[B29] Blum K Braverman ER Holder JM Lubar JF Monastra VJ Miller D Lubar JO Chen TJ Comings DE Reward deficiency syndrome: a biogenetic model for the diagnosis and treatment of impulsive, addictive, and compulsive behaviors J Psychoactive Drugs 2000 32 1 112 10.1080/02791072.2000.10736099 11280926

[B30] Torrens M Serrano D Astals M Perez-Dominguez G Martin-Santos R Diagnosing comorbid psychiatric disorders in substance abusers: validity of the Spanish versions of the Psychiatric Research Interview for Substance and Mental Disorders and the Structured Clinical Interview for DSM-IV Am J Psychiatry 2004 161 1231 1237 10.1176/appi.ajp.161.7.1231 15229056

[B31] Caton CL Drake RE Hasin DS Dominguez B Shrout PE Samet S Schanzer B Differences between early phase primary psychotic disorders with concurrent substance Arch Gen Psychiatry 2005 62 137 145 10.1001/archpsyc.62.2.137 15699290

[B32] Schuckit MA Saunders JB The empirical basis of substance use disorders diagnosis: research recommendations for the Diagnostic and Statistical Manual of Mental Disorders, fifth edition (DSM-V) Addiction 2006 101 170 173 10.1111/j.1360-0443.2006.01611.x 16930174

[B33] Brady K Casto S Lydiand RB Malcolm R Arana G Substance abuse in an inpatient psychiatric sample Am J Drug Alcohol Abuse 1991 17 389 397 10.3109/00952999109001598 1746501

[B34] Satel SL Edell WS Cocaine-induced paranoia and psychosis proneness Am J Psychiatry 1991 148 1708 1711 1957934 10.1176/ajp.148.12.1708

[B35] Bartlett E Hallin A Chapman B Angrist B Selective sensitization to the psychosis-inducing effects of cocaine: a possible marker for addiction relapse vulnerability? Neuropsychopharmacology 1997 16 77 82 10.1016/S0893-133X(96)00164-9 8981391

[B36] Griffith JD Costa E, Garattini S Experimental psychosis induced by the administration of d-amphetamine Amphetamine and Related Compounds 1970 New York, USA: Raven Press 876 904

[B37] Angrist BM Gershon S The phenomenology of experimentally induced amphetamine psychosis. Preliminary observations Biol Psychiatry 1970 2 95 107 5459137

[B38] Bell DS The experimental reproduction of amphetamine psychosis Arch Gen Psychiatry 1973 29 35 40 4711131 10.1001/archpsyc.1973.04200010020003

[B39] Janowsky DS Risch C Amphetamine psychosis and psychotic symptoms Psychopharmacology 1979 65 73 77 10.1007/BF00491982 116294

[B40] Angrist B Cho AK, Segal DS Amphetamine psychosis: clinical variations of the syndrome Amphetamine and its Analogs 1995 New York, USA: Academic Press

[B41] Kalayasiri R Sughondhabirom A Gueorguieva R Coric V Lynch WJ Morgan PT Cubells JF Malison RT Self-reported paranoia during laboratory "binge" cocaine self administration in humans Pharmacol Biochem Behav 2006 83 249 256 10.1016/j.pbb.2006.02.005 16549106

[B42] Thomas H A community survey of adverse effects of cannabis use Drug Alcohol Depend 1996 42 201 207 10.1016/S0376-8716(96)01277-X 8912803

[B43] Chaudry HR Moss HB Bashir A Suliman T Cannabis psychosis following bhang ingestion Br J Addict 1991 86 1075 1081 10.1111/j.1360-0443.1991.tb01874.x 1932878

[B44] Solomons K Neppe VM Kuyl JM Toxic cannabis psychosis is a valid entity S Afr Med J 1990 78 476 481 2218786

[B45] Wylie AS Scott RTA Burnett SJ Psychosis due to 'skunk' BMJ 1995 311 125 7613377 10.1136/bmj.311.6997.125 PMC2550170

[B46] Hall W Degenhardt L Teesson M Cannabis use and psychotic disorders: an update Drug Alcohol Rev 2004 23 433 443 10.1080/09595230412331324554 15763748

[B47] Tien AY Anthony JC Epidemiological analysis of alcohol and drug use as risk factors for psychotic experiences J Nerv Ment Dis 1990 178 473 480 2380692

[B48] Tsuang JW Lohr JB Effects of alcohol on symptoms in alcoholic and nonalcoholic patients with schizophrenia Stud Alcohol 2002 63 145 155 10.1176/ps.45.12.1229 7868108

[B49] Levinson I Rosenthal RN Methadone withdrawal psychosis J Clin Psychiatry 1995 56 73 76 7852256

[B50] Maremmani I Pani PP Canoniero S Pacini M Perugi G Rihmer Z Akiskal HS Is the bipolar spectrum the psychopathologic substrate of suicidality in heroin addicts? Psychopathology 2007 40 269 277 10.1159/000104742 17622705

[B51] Grant S London ED Newlin DB Villemagne VL Liu X Contoreggi C Phillips RL Kimes AS Margolin A Activation of memory circuits during cue-elicited cocaine craving Proc Natl Acad Sci USA 1996 93 12040 12045 10.1073/pnas.93.21.12040 8876259 PMC38179

[B52] Maas LC Lukas SE Kaufman MJ Weiss RD Daniels SL Rogers VW Kukes TJ Renshaw PF Functional magnetic resonance imaging of human brain activation during cue-induced cocaine craving Am J Psychiatry 1998 155 124 126 9433350 10.1176/ajp.155.1.124

[B53] Volkow ND Fowler JS Wolf AP Hitzemann R Dewey S Bendriem B Alpert R Hoff A Changes in brain glucose metabolism in cocaine dependence and withdrawal Am J Psychiatry 1991 148 621 626 2018164 10.1176/ajp.148.5.621

[B54] Breiter HC Gollub RL Weisskoff RM Kennedy DN Makris N Berke JD Goodman JM Kantor HL Gastfriend DR Riorden JP Mathew RT Rosen BR Hyman SE Acute effects of cocaine on human brain activity and emotion Neuron 1997 19 591 611 10.1016/S0896-6273(00)80374-8 9331351

[B55] Volkow ND Wang GJ Fowler JS Hitzemann R Angrist B Gatley SJ Logan J Ding YS Pappas N Association of methylphenidate-induced craving with changes in right striato-orbitofrontal metabolism in cocaine abusers: implications in addiction Am J Psychiatry 1999 156 19 26 9892293 10.1176/ajp.156.1.19

[B56] Wexler BE Gottschalk CH Fulbright RK Prohovnik I Lacadie CM Rounsaville BJ Gore JC Functional magnetic resonance imaging of cocaine craving Am J Psychiatry 2001 158 86 95 10.1176/appi.ajp.158.1.86 11136638

[B57] Volkow ND Wang GJ Ma Y Fowler JS Zhu W Maynard L Telang R Vaska P Ding YS Wong C Swanson JM Expectation enhances the regional brain metabolic and the reinforcing effects of stimulants in cocaine abusers J Neurosci 2003 23 11461 11468 14673011 10.1523/JNEUROSCI.23-36-11461.2003 PMC6740524

[B58] Bickel WK Odum AL Madden GJ Impulsivity and cigarette smoking: Delay discounting in current, never, and ex-smokers Psychopharmacology 1999 146 447 454 10.1007/PL00005490 10550495

[B59] Fillmore MT Rush CR Impaired inhibitory control of behavior in chronic cocaine users Drug Alcohol Depend 2002 66 265 273 10.1016/S0376-8716(01)00206-X 12062461

[B60] Lejuez CW Aklin WM Jones HA Richards JB Strong DR Kahler CW Read JP The balloon analogue risk task (BART) differentiates smokers and nonsmokers Exp Clin Psychopharmacol 2003 11 26 33 10.1037/1064-1297.11.1.26 12622341

[B61] Madden GJ Petry NM Badger GJ Bickel WK Impulsive and self control choices in opioid dependent patients and non-drug-using control participants: drug and monetary rewards Exp Clin Psychopharmacol 1997 5 256 262 10.1037/1064-1297.5.3.256 9260073

[B62] Mitchell S Measures of impulsivity in cigarette smokers and non-smokers Psychopharmacology 1999 146 455 464 10.1007/PL00005491 10550496

[B63] Petry NM Casarella T Excessive discounting of delayed rewards in substance abusers with gambling problems Drug Alcohol Depend 1999 56 25 32 10.1016/S0376-8716(99)00010-1 10462089

[B64] Reynolds B Richards JB Horn K Karraker K Delay discounting and probability discounting as related to cigarette smoking status in adults Behav Process 2004 65 35 42 10.1016/S0376-6357(03)00109-8 14744545

[B65] Vuchinich RE Simpson CA Hyperbolic temporal discounting in social drinkers and problem drinkers Exp Clin Psychopharmacol 1998 6 292 305 10.1037/1064-1297.6.3.292 9725113

[B66] Dawes MA Tarter RE Kirisci L Behavioral self-regulation: correlates and 2 year follow-ups for boys at risk for substance abuse Drug Alcohol Depend 1997 45 165 176 10.1016/S0376-8716(97)01359-8 9179518

[B67] Moeller FG Dougherty DM Barratt ES Oderinde V Mathias CW Harper RA Swann AC Increased impulsivity in cocaine dependent subjects independent of antisocial personality disorder and aggression Drug Alcohol Depend 2002 68 105 111 10.1016/S0376-8716(02)00106-0 12167556

[B68] Tarter RE Kirisci L Mezzich A Cornelius JR Pajer K Vanyukov M Gardner W Blackson T Clark D Neurobehavioral disinhibition in childhood predicts early age at onset of substance use disorder Am J Psychiatry 2003 160 1078 1085 10.1176/appi.ajp.160.6.1078 12777265

[B69] Tarter RE Kirisci L Habeych M Reynolds M Vanyukov M Neurobehavior disinhibition in childhood predisposes boys to substance use disorder by young adulthood: direct and mediated etiologic pathways Drug Alcohol Depend 2004 73 121 132 10.1016/j.drugalcdep.2003.07.004 14725951

[B70] Clark DB Cornelius JR Kirisci L Tarter RE Childhood risk categories for adolescent substance involvement: a general liability typology Drug Alcohol Depend 2005 77 13 21 10.1016/j.drugalcdep.2004.06.008 15607837

[B71] Kirisci L Tarter RE Reynolds M Vanyukov M Individual differences in childhood neurobehavior disinhibition predict decision to desist substance use during adolescence and substance use disorder in young adulthood: a prospective study Addict Behav 2006 31 686 696 10.1016/j.addbeh.2005.05.049 15964148

[B72] Nigg JT Wong MM Martel MM Jester JM Puttler LI Glass JM Adams KM Fitzgerald HE Zucker RA Poor response inhibition as a predictor of problem drinking and illicit drug use in adolescents at risk for alcoholism and other substance use disorders J Am Acad Child Adolesc Psychiatry 2006 45 468 475 10.1097/01.chi.0000199028.76452.a9 16601652

[B73] Petry NM Delay discounting of money and alcohol in actively using alcoholics, currently abstinent alcoholics, and controls Psychopharmacology 2001 154 243 250 10.1007/s002130000638 11351931

[B74] Reynolds B Do high rates of cigarette consumption increase delay discounting? A cross-sectional comparison of adolescent smokers and young-adult smokers and nonsmokers Behav Process 2004 67 545 549 10.1016/j.beproc.2004.08.006 15519004

[B75] Bornovalova MA Daughters SB Hernandez GD Richards JB Lejuez CW Differences in impulsivity and risk-taking propensity between primary users of crack cocaine and primary users of heroin in a residential substance-use program Exp Clin Psychopharmacol 2005 13 311 318 10.1037/1064-1297.13.4.311 16366761

[B76] Reynolds B The Experiential Discounting Task is sensitive to cigarette-smoking status and correlates with a measure of delay discounting Behav Pharmacol 2006 17 133 42 10.1097/01.fbp.0000190684.77360.c0 16495721

[B77] Vassileva J Gonzalez R Bechara A Martin EM Are all drug addicts impulsive? Effects of antisociality and extent of multidrug use on cognitive and motor impulsivity Addict Behav 2007 32 3071 3076 10.1016/j.addbeh.2007.04.017 17507173 PMC2128047

